# Impact of Oxidative Stress on Molecular Mechanisms of Cervical Ripening in Pregnant Women

**DOI:** 10.3390/ijms232112780

**Published:** 2022-10-24

**Authors:** Maciej W. Socha, Wojciech Flis, Mateusz Wartęga, Martyna Stankiewicz

**Affiliations:** 1 Department of Perinatology, Gynecology and Gynecologic Oncology, Faculty of Health Sciences, Collegium Medicum in Bydgoszcz, Nicolaus Copernicus University, Łukasiewicza 1, 85-821 Bydgoszcz, Poland; 2 Department of Obstetrics and Gynecology, St. Adalbert’s Hospital in Gdańsk, Copernicus Healthcare Entity, Jana Pawła II 50, 80-462 Gdańsk, Poland; 3 Department of Pathophysiology, Faculty of Pharmacy, Collegium Medicum in Bydgoszcz, Nicolaus Copernicus University, M. Curie-Skłodowskiej 9, 85-094 Bydgoszcz, Poland

**Keywords:** oxidative stress, reactive nitrogen species, reactive oxygen species, RNS, ROS, cervical ripening, pregnancy, gestation, cervix

## Abstract

Uterine cervix is one of the essential factors in labor and maintaining the proper course of pregnancy. During the last days of gestation, the cervix undergoes extensive changes manifested by transformation from a tight and rigid to one that is soft and able to dilate. These changes can be summarized as “cervical ripening”. Changes in the cervical tissue can be referred to as remodeling of the extracellular matrix. The entire process is the result of a close relationship between biochemical and molecular pathways, which is strictly controlled by inflammatory and endocrine factors. When the production of reactive oxygen species exceeds the antioxidant capacity, oxidative stress occurs. A physiologic increase of reactive oxygen species (ROS) and reactive nitrogen species (RNS) is observed through pregnancy. ROS play important roles as second messengers in many intracellular signaling cascades contributing to the course of gestation. This review considers their involvement in the cervical ripening process, emphasizing the molecular and biochemical pathways and the clinical implications.

## 1. Introduction

The uterine cervix is one of the most crucial factors in labor and maintaining the proper course of pregnancy. Over a normal course of gestation, the cervix provides an immune and physical barrier to the unimpeded development of the fetus. However, during the last days of pregnancy cervix undergoes extensive changes. Those changes are manifested by transformation from a firm, tough tissue to one that is gentle and prone to dilation. These changes result from a series of complex biochemical pathways leading to the rearrangement of the cervical extracellular matrix. The whole of this complex process occurring in cervical connective tissue during term pregnancy can be summarized in the term “cervical ripening.” In turn, the cervix transforms from closed and rigid to soft and susceptible, which allows the cervix to pass the presenting part of the fetus. Subsequently, due to systolic uterine activity, the cervix can dilate and pass the presenting part of the fetus. Despite the tremendous research conducted over the years, the entire cervical ripening process still seems poorly understood. The role of specific regulatory factors involved in cervical ripening has been described. Recently, an increasingly important role (both in the physiology and pathophysiology of pregnancy) has been assigned to oxidative stress and reactive oxygen species.

Oxidative stress (OS) and the production of reactive oxygen and nitrogen species in the fetal compartments can contribute to various physiological processes during pregnancy. A small amount of reactive oxygen species is required for the proper functioning of the cell. Reactive oxygen species (in tandem with other regulatory factors) are one of the major components that are responsible for maintaining proper pregnancy homeostasis. However, excessive production of ROS can overwhelm the antioxidant defense system, damaging lipids, proteins, and DNA which leads to cellular damage that contributes to dysfunctional tissue. ROS increases significantly in the fetal compartments at term, which promotes a number of changes that occur in the maternal compartment [1]. The effects of OS on the tissues of the fetus and mother have been thoroughly investigated. However, it is not fully understood what effect oxidative stress and reactive oxygen species may have on cervical tissue. In this review, we discuss the role of OS in regulating the cervical ripening process.

## 2. Anatomy and Physiology of Cervix

The human cervix is the inferior part of the uterus and can be divided into the supravaginal and lower portions. The cervical canal, lined with a cylindrical epithelium containing a large number of glands, runs between the internal and external os. The epithelium contains a monolayer of cells that produces a complex mucus of water, ions, enzymes, plasma proteins, and mucin glycoproteins [2]. During pregnancy, the epithelium and glands proliferate significantly. The glands produce dense mucus that forms a plug that fills the cervical canal. The plug closes the external os of the cervix until delivery, creating a physical and immunological barrier to the unimpeded development of the fetus [3]. The cervix is formed by connective tissue, smooth muscle, and the ground substance infiltrated by blood vessels [4]. The cellular element consists of fibroblasts, wandering cells, and mast cells.

Additionally, during the inflammation that develops during labor, a large influx of inflammatory cells can be observed [5]. The main component of the cervix is connective tissue with approximately 10–15% of smooth muscle [6]. In addition, a small number of elastin fibers cross-linked into microfibrils can be found in the cervix [7]. Smooth muscles are located mainly around the cervical internal os and are circumferentially organized in a sphincter-like pattern. The rest of the smooth muscle cells are randomly located in the cervical stroma [8]. Particularly noteworthy is the structure of the extracellular matrix (ECM). It contains a large amount of collagen—mainly type I [9]. Collagen fibers are linked by covalent bonds. The cervical stroma consists of glycosaminoglycans (GAGs) whose chains form covalent bonds with the protein core—creating proteoglycans that have the ability to bind collagen fibers [10]. They include hyaluronan (HA), dermatan sulfate, heparan sulfate, and chondroitin sulfate. This type of structure of the cervical tissue creates a highly coherent structure which makes it stable and contributes significantly to the stiffness of the cervical structure [11].

**Figure 1:**
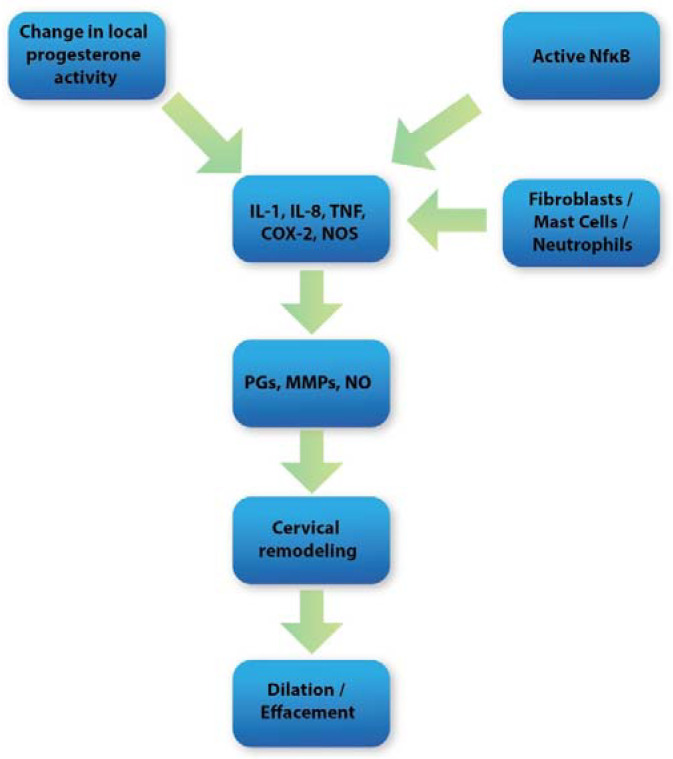
Diagram showing overall molecular mechanisms during cervical ripening.

As mentioned before, the cervix undergoes significant changes in the last days of pregnancy, called cervical ripening. These changes are a complex process derived from enzymatic degradation, inflammation, and endocrine regulation [9,12]. The main biochemical events during cervical ripening are collagen and elastin fibers degradation, increased fluid intake, changes in glycosaminoglycans quality with increased hyaluronan synthesis, and inflammatory response mediated by the increased distribution of inflammatory cells [13,14]. However, the most marked change is the reduction in total collagen concentration in cervical tissue, achieved through lessening expression of collagen assembly genes and increased expression of matrix metalloproteinases (MMPs) that degrade collagen fibers. As a result, collagen fibers lose their tightly packed structure and become more widely spread out, which makes the cervix lose its rigidity. Moreover, MMPs can digest other ECM compounds, such as proteoglycans, laminin, and fibronectin [15,16,17,18] (Figure 1).

Cervical ripening corresponds in many aspects to the inflammatory process. Local vasodilatation associated with increased vascular permeability with water inflow and inflammatory cell influx can be observed [19,20]. The presence of inflammatory cells such as mast cells, macrophages, and neutrophils is pivotal. They mainly secrete proinflammatory cytokines, nitric oxide, and prostaglandins, key players involved in cervical ripening [21,22,23,24,25,26,27]. Activation of immune cells is not a random event but is mediated by both paracrine and endocrine factors. The entire process of cervical remodeling is strictly regulated, which we will discuss later in this review.

## 3. Oxidative Stress

The term “reactive oxygen species (ROS)” mainly refers to free radicals, which are defined as molecules that contain unpaired electrons, which give them tremendous reactivity. This reactivity is possible due to the presence of an unpaired electron in the outer shell of the atom. Free radicals can be generated from various elements, but in human homeostasis, the dominant forms are those with oxygen and nitrogen [28]. Under normal conditions, they are constantly produced in small amounts in many cellular compartments, such as the mitochondrial respiratory chain and the endoplasmic reticulum. Under normal conditions, they are constantly produced in small amounts by the endoplasmic reticulum and in the respiratory chain. During physiological conditions, ROS actively participate in cell signaling as a second messenger via activation of protein kinases, opening of ion channels, activation of transcription factors, apoptosis, and protein modifications [29,30]. However, under pathological conditions, the presence of excess ROS leads to cellular DNA damage, lipids, and cellular structure breakage. The main free radicals are superoxide anion, hydrogen peroxide, peroxynitrite (ONOO^−^), and hydroxyl ion. The most reactive oxygen free radical is superoxide anion. The main source of superoxide anion is the mitochondrial respiratory chain. The respiratory chain is made up of four multimeric complexes (I–IV), cytochrome C, and coenzyme Q [31]. During the mitochondrial oxidative phosphorylation process maintained by those complexes, the adenosine triphosphate (ATP) is formed as a result of the transfer of electrons to O_2_ from NADH [32,33]. The transfer of electrons through (during oxidative phosphorylation) is not fully effective and electron leakage occurs periodically onto molecular oxygen, resulting in the formation of superoxide anion. The electron leakage through the respiratory chain may vary depending on local oxygen conditions. A noticeable increase in the creation of superoxide anion in hypoxia and hyperoxia conditions can be observed [34]. Physiologically, almost 2% of oxygen is converted into superoxide in mitochondria [28]. The other physiological sources of the superoxide anion that constantly produce it in small amounts are endoplasmic reticulum, cytochrome P450, and nicotinamide adenine dinucleotide phosphate (NADPH) oxidase [35]. 

Reactive nitrogen species include nitric oxide (NO), which is a pivotal regulatory molecule, especially in the cervical ripening process [9], nitrogen dioxide (NO_2_), nitrosamines, and peroxynitrite (ONOO^−^) [36]. NO is synthesized in the reaction mediated by nitric oxide synthase (NOS), which uses NADPH as an electron donor. Three isoforms of this enzyme have been isolated. These include: neuronal NOS (nNOS), inducible NOS (iNOS) and endothelial NOS (eNOS [37,38]). NO has strong vasodilatory properties and is crucial for maintaining the proper cervical ripening process. Although the effects of NO may be crucial for proper homeostasis, excessive production of NO can affect enzyme activity and cell signaling [39]. 

The imbalance between pro-oxidant and antioxidant molecules can cause oxidative stress (OS). The ratio of the molecules can be altered by a decrease in antioxidant mechanisms or an increment in the concentration of ROS or RNS. In both situations, an accumulation of free radicals can be observed. As mentioned above, a certain amount of ROS is required to maintain proper cell functioning. However, excessive ROS synthesis may overcome the antioxidant defense system and create an inadequate environment for normal cell functioning [28,40,41].

Intracellular ROS production is controlled by highly complex and integrated antioxidant systems. These include enzymatic defenses, such as superoxide dismutase and glutathion peroxidase, and non-enzymatic defenses which are mostly vitamins such as vitamins E and C that directly scavenge ROS [30,32].

Oxidative stress occurs at the maternal-fetal interface from early pregnancy onwards, gradually increasing at the term [42]. An increase in ROS concentration at term is achieved mainly due to increased metabolic demands with the increase in oxygen consumption, elevated tissue oxygen requirements, and reduction in substrate supply [28,32,34,35,36]. Additionally, a decrease in total antioxidant capacity, including levels of superoxide dismutase, thioredoxin-1, zinc, and copper, the concentration of which in the cervical fluid gradually decreases during labor, can be observed [32].

In the following sections of this publication, we will describe the influence of oxidative stress and ROS on the individual components of the cervical ripening process.

## 4. Oxidative Stress and Apoptosis

Apoptosis is an essential process that eliminates undesired cells. It is a regulated process of cell self-destruction, which is crucial for maintaining proper homeostasis. It is a process presenting biochemical features such as condensation of chromatin and nuclear fragmentation, cell shrinking, and membrane blebbing. Subsequently, apoptotic bodies are produced, which are digested by macrophages. This results in the elimination of the cell [43].

Apoptosis is largely mediated by caspases which are endoproteases that hydrolyze peptide bonds in the substrate. Caspases are initially produced as zymogens that require cleavage for activation [44].

There are at least 1000 substrates for caspases, which can be found in the cell compartment. These include cell cycle regulation proteins, structural proteins, and DNA [45]. In addition, caspases can disrupt the electron transport chain by cleaving the p75 subunit of complex I in the mitochondria, which disrupts oxidative phosphorylation and hence the critical function of ATP production [46,47]. Apart from influencing the functioning of the cell, caspases have the ability to interfere with the cell structure by affecting actin polymerization [48]. As a result, the cytoskeleton is improperly organized. In addition to the direct influence of caspases on the structure and function of cells, they also take an active part in inflammation by forming an inflammasome. Activation and assembly of the inflammasome promote the secretion of proinflammatory cytokines such as interleukin-1 (IL-1) which is actively involved in triggering an inflammatory response [46,49]. It seems that apoptosis may also influence the inflammatory response, which is a key player in the proper cervical ripening process.

Apoptosis is regulated by 3 major pathways, such as the mitochondrial, endoplasmic, and extrinsic pathways [46]. ROS can, both directly and indirectly, stimulate each of these pathways [50]. The mitochondrial pathway is regulated by the permeability of the inner mitochondrial membrane, which is physiologically impermeable. Under excessive ROS production, the inner membrane permeability increases [51,52]. This, in turn, allows protons to flow into the mitochondrial matrix, disrupting the oxidative phosphorylation and osmotic swelling of the mitochondrial matrix, resulting in the rupture of the mitochondrial membrane. Together with increased outer mitochondrial membrane permeabilization, this leads to the release of cytochrome c into the cytosol, which is crucial for apoptosome formation [52,53,54]. In turn, active apoptosome activates other caspases. Reactive oxygen species cause the oxidation of cardiolipin, which causes the release of cytochrome c into the cytosol and the formation of the apoptosome. In addition, ROS causes depolarization and opening of the mitochondrial membrane, which disrupts oxidative phosphorylation. Secondly, ROS can activate the p53 N-terminal kinase and/or c-Jun (JNK), which activate pro-apoptotic Bcl-2 proteins localized in the outer mitochondrial membrane that inhibit the function of anti-apoptotic proteins and further enhance the permeability of the inner mitochondrial membrane. Finally, ROS can directly cause mitochondrial DNA (mtDNA) fragmentation [48,50,51,53].

Another apoptotic pathway significantly influenced by reactive oxygen species is the endoplasmic pathway, which is significantly associated with calcium ions. There is a great relationship between calcium ions and the proper functioning of the mitochondria. Excessive accumulation of mitochondrial Ca^2+^ can increase the permeability of the mitochondrial membrane [55]. This leads to osmotic swelling and causes the rupture of the mitochondrial membrane, which leads to the release of cytochrome c into the cytosol. Excessive ROS production can directly stimulate the release of calcium ions from the ER [28]. Then calcium ions are captured by the mitochondria, which initiates apoptosis.

The last but extremely important pathway of apoptosis activation is the extrinsic pathway mediated by the tumor necrosis factor receptors (TNF-R), which are located in the cell membrane. Activation of these receptors causes the activation of caspases and stimulate the release of cytochrome c into the cytosol where it participates in the formation of the apoptosome [50,56,57,58,59]. Furthermore, these receptors are stimulated by excessive ROS production, triggering apoptosis [49,50]. Summing up, reactive oxygen species have the ability to stimulate apoptosis at almost every stage of it, influencing, both directly and indirectly, all the elements of individual signaling pathways.

As mentioned, reactive oxygen species (the activity and concentration of which both increase significantly during pregnancy) can cause cell damage that contributes to cell rearrangement. Apart from the remodeling of the cervical tissue during its maturation, apoptosis of cervical cells also takes place with a noticeable increase at term pregnancy [60]. We believe that oxidative stress may contribute to cervical cell apoptosis. Interestingly, treatment of the cervical cells with cigarette smoke extract, an oxidative stress inducer, triggered ROS production promoting cell cycle arrest and significantly induced apoptosis [61]. Moreover, it turns out that in term pregnancy, the increment of the expression of glutaredoxin in the cervical tissue can be observed [62]. Glutaredoxin, a member of the thioredoxin superfamily, plays a key role in the delivery of electrons to ribonucleotide reductase, which is essential for DNA repair and is an important antioxidant factor. It appears that oxidative stress, which occurs in cervical tissue at term, may affect redox potential, which in turn leads to increased expression of glutaredoxin. Taking all the above into consideration, we believe that reactive oxygen species are strongly involved in cervical ripening by direct stimulation of apoptosis of cervical cells and induction of the expression of antioxidants. 

## 5. Oxidative Stress and Inflammation in Cervical Tissue

**Figure 2:**
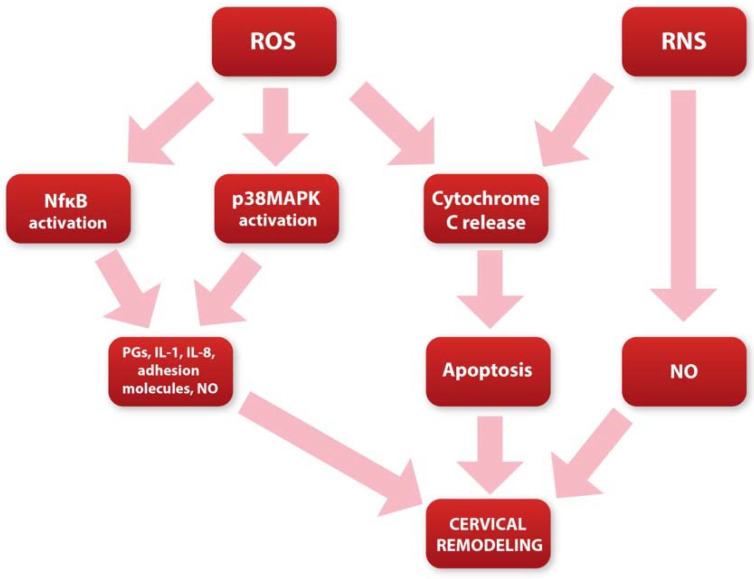
Diagram showing potential role of ROS and RNS in cervical ripening.

The physiological changes that occur during cervical ripening largely correspond to the inflammatory process. Many of the mediators and enzymes involved in the regulation of the acute inflammatory response are also strongly involved in the regulation of the cervical ripening process [19]. Local vasodilatation associated with increased vascular permeability occurs during cervical ripening. This, in turn, leads to intensified water inflow and inflammatory cell influx. Subsequently, a large presence of neutrophils, mast cells, and macrophages in cervical stroma arises at term [20]. The role of these cells is to secrete prostaglandins (PGs), metalloproteinases (MMPs), adhesion molecules, nitric oxide (NO), and proinflammatory cytokines such as IL-8, IL-8, IL-1, and TNF, which are among the most crucial mediators responsible for the proper course of cervical ripening [9]. It seems that ROS may be closely related to the mentioned factors in inflammation occurring during cervical ripening. During pregnancy, the ROS-inflammatory axis works in parallel (Figure 2) in a balanced system maintained by anti-inflammatory mediators and antioxidants. However, imbalance in this process occurs at term leading to the molecular and biochemical changes in cervical tissue [63]. 

Interleukin 1 (IL-1) is a powerful cervical ripening regulator. Vaginal application of suppositories with IL-1 leads to significant softening of the cervix [64]. IL-1 has the ability to downregulate PG dehydrogenase (PGDH) and upregulate cyclooxygenase-2 (COX-2) which are pivotal enzymes involved in the synthesis and degradation of prostaglandins. Thus, due to IL-1 action, there is a significant increment in PGs concentration in cervical tissue at term [65]. Prostaglandins, in turn, can directly and indirectly affect cervical ripening by stimulating the expression of endothelial adhesion molecules and IL-8, increasing water content and lessening total collagen concentration [66]. Additionally, IL-1 stimulates the secretion of other proinflammatory cytokines, such as IL-8, IL-6, and TNF. Finally, IL-1 enhances the expression of MMPs and inhibits the action of TIMPs (tissue inhibitors of metalloproteinase), leading to the multiplication of collagenolytic activity in cervical tissue [67]. 

IL-8, the synthesis of which is greatly enhanced during term pregnancy mainly due to monocytes, neutrophils, and fibroblasts action, significantly impacts the remodeling of the cervical tissue [68]. IL-8 is a strong neutrophil chemotactic agent. It actively participates in inflammatory response by affecting vascular permeability leading to the leukocyte influx. Interleukin-8 not only induces the migration of neutrophils to the cervical stroma but also promotes the release of neutrophil proteases such as MMPs [69]. IL-1 and IL-8 appear to be closely related by directly increasing IL-8 expression by IL-1 in cervical tissue [70].

The last but not least potent paracrine mediator of inflammatory functions is TNF-α (tumor necrotic factor), which is secreted mainly by macrophages. It has the ability to directly stimulate the synthesis of MMPs and PGs [71]. Apart from its role in the development of inflammation, it may actively participate in triggering apoptosis by acting on the extrinsic apoptosis pathway mediated by TNF-R superfamily membrane receptors. 

MAPKs (mitogen-activated protein kinases) are proline-directed serine and threonine protein kinases. p38 proteins which is a subtype of MAPKs are major players during an inflammatory reaction. They are strongly expressed especially in macrophages whose concentration increases in the cervical stroma during cervical ripening. The p38 family can be divided into four subtypes: α, *β*, *γ*, and *δ*. The p38 kinases are activated by a variety of factors, including cytokines and TLR ligands [72]. Those factors trigger the phosphorylation of p38, leading to the activation and translocation of p38MAPK to the nucleus and, in turn, activation of transcription of inflammatory-associated genes [73,74]. Evidence suggests that p38MAPK is strongly involved in the macrophage-mediated inflammatory response [72]. This involvement manifests itself through increased expression of proinflammatory cytokines, PGE2, and COX-2. Additionally, p38 enhances the secretion of endothelial vascular cell adhesion molecule—1 (VCAM-1), which is a key adhesion molecule involved in leukocyte influx during inflammation [61,72]. Recent studies show unequivocally that ROS can directly activate p38MAPKs in cervical tissue and thus enhance the inflammatory response [61,75,76]. In addition, activation of p38MAPK leads to a direct increment in the secretion of metalloproteinases (MMPs), particularly MMP-9, which is a crucial factor required for the enzymatic breakdown of collagen fibers in the cervical stroma [76,77]. Moreover, it turns out that proinflammatory cytokines can increment the production of ROS. Cytokines, especially IL-1 and TNF-α whose concentration significantly increases during cervical ripening, can directly stimulate mitochondrial- and NADPH oxidase-generated reactive oxygen species during inflammation [78]. In turn, free radicals recruit inflammatory mediators such as p38MAPK, which leads to the amplification of sterile inflammation occurring in the cervical stroma [79]. 

The role of NF-κB (Nuclear Factor-kappaB) in cervical ripening has been known for some time. Its involvement is mainly manifested by the intensification of the inflammatory response in the cervical stroma. NF-κB is heavily influenced by inflammatory factors and endocrine factors such as glucocorticoids and progesterone, which play a regulatory role in cervical ripening [80,81]. Additionally, it turns out that oxidative stress (OS) may also trigger NF-κB activation. NF-κB is a family of transcription factors such as cRel, p50, p52, and RelA/p65, RelB. In its inactive form, NF-κB forms a complex with NF-κB inhibitor, which is expressed in 3 isoforms such as IkBα, IkBβ, and IkBε. These three isoforms have the ability to inactivate specific NF-κB subunits. These inhibitors are regulated by IκB kinase (IKK), which is a protein complex. IKK can phosphorylate IkBα subsequently leading to IkBα degradation and allowing NF-κB to activate [82,83]. Activated NF-κB gains the ability to translocate to the nucleus and subsequently activate gene transcription, thus enhancing the expression of inflammatory mediators such as IL-1, IL-8, IL-6, iNOS, and COX-2 which are crucial for cervical ripening [84]. As mentioned above, IL-1 and TNF can stimulate ROS production by enhancing the activity of neutrophil NADPH oxidase. The excess of ROS produced by NADPH oxidase can directly activate protein kinase B (PKB) and NIK (NF-κB inducing kinase), which phosphorylate IKKα. As a result, NF-κB becomes activated, and the expression of factors regulating cervical remodeling is stimulated [73,85,86,87,88]. 

Taking all the above into consideration, we believe that ROS are factors strongly influencing the development of sterile inflammation in cervical tissue. By showing an effect on both p38MAPK and NF-κB activity, they can actively contribute to cervical ripening. However thorough understanding of this phenomenon and determining the influence of other factors on NF-κB and p38MAPK activity require further research.

## 6. Reactive Nitrogen Species and Cervical Ripening

While technically nitric oxide (NO) is actually affiliated with the nitrogen reactive species (RNS) group, we mention it here because NO is highly produced in materno-fetal compartments where it contributes strongly to both cervical ripening and the proper course of pregnancy. Additionally, NO can form the peroxynitrite (ONOO^−^) which is a highly reactive agent. Therefore, NO can contribute to oxidative stress [82]. 

NO is synthesized from L-arginine. This reaction is mediated by three nitric oxide synthases (NOS), which include: inducible NOS (iNOS), neuronal NOS (nNOS), and endothelial NOS (eNOS). The expression of iNOS is induced by LPS and cytokines without the involvement of calcium and produces large quantities of NO over time. Whereas nNOS and eNOS activity is calcium-dependent and their expression is constitutional [37]. NO can act as an intracellular messenger, a paracrine mediator, and a neurotransmitter affecting target tissues directly and indirectly. The direct effect is manifested by stimulating guanylate cyclase, which stimulates the conversion of GTP to cGMP. The indirect effect includes oxidation and nitration which can lead to altered protein structure and function. Each NOS can be found in the cervical tissue. However, the dominant form is iNOS. An increase in the concentration of NOSs with a subsequent increase in NO production during human labor can be observed in the cervical tissue. Macrophages and neutrophils are the main source of NO in the cervical stroma, as they express iNOS [89,90]. There is ample evidence that NO is strongly involved in the cervical ripening process. A local administration of sodium nitroprusside, a NO donor, can effectively induce cervical ripening while not inducing labor in pregnant guinea pigs [91]. Moreover, treatment with a NOS inhibitor significantly delayed cervical ripening, which resulted in prolonged deliveries [92]. The effect of nitric oxide on the cervix is manifested mainly by enhancing the activity of metalloproteinases (mainly MMP-9 and MMP-2), which cleave collagen cross-links [93,94]. Additionally, NO can promote leukocyte influx in cervical stroma by inducing local vasodilatation and stimulating IL-8 production, which is a powerful neutrophil chemotactic factor [95,96]. Finally, NO is a puissant prostaglandin synthesis inducer by stimulating COX-2 expression [96,97]. Moreover, it seems that PGs can induce NO release in cervical tissue as well—a local application of misoprostol, prostaglandin E1 analog, enhances NOS expression in the cervical stroma [98,99]. 

Nitric oxide can exhibit effects on the myometrium and uteroplacental circulation. In human uteroplacental circulation, NO stimulates the production of cGMP, which directly relaxes both placental and umbilical vessels, causing a significant decrease in vascular flow resistance [100,101]. Interestingly, it appears that nitric oxide may be closely related to progesterone. Progesterone, which is high during pregnancy, may influence the expression of nitric oxide synthase in various compartments. During the proper course of gestation, due to progesterone action, cervical NO synthesis is suppressed, and uterine NO production is stimulated, leading to the inhibition of cervical ripening, and maintaining uterine quiescence. In turn, when local progesterone activity significantly decreases at term, uterine NO production is diminished, and cervical nitric oxide synthesis rises. Thus, inducing the cervical ripening process [91,102,103,104]. 

Interestingly, nitric oxide may also be actively involved in apoptosis. Nitric oxide, which is overproduced during cervical ripening, can act as a pro-apoptotic modulator, stimulating cytochrome c transfer to the cytosol, subsequently leading to the activation of caspase pathways [105,106]. 

As mentioned above, NF-κB is a strong transcription factor that can be activated by a variety of factors. NF-κB dependent genes in cervical tissue include products that promote cervical ripening, including proinflammatory cytokines, COX-2, and iNOS. It is evident that NF-κB stimulates the production of iNOS, which increases the production of nitric oxide. However, it appears that NO may also influence the activity of NF-κB. Studies have shown that NO can sustainably keep NF-κB in an active state. It is achieved most likely by generating amounts of peroxynitrite (ONOO^−^). As a result, the NF-κB nuclear translocation is permanently maintained, and thus the continuous transcription of NF-κB dependent genes is ensured [107]. This may suggest the presence of a regulatory loop, where NF-κB increases the synthesis of nitric oxide. Subsequently, nitric oxide sustains continuous NF-κB activation, stimulating the production of factors involved in the cervical remodeling process. However, a detailed understanding of the relationship between these two substances requires further research.

## 7. Discussion

The overall aim of this review is to present a current understanding of the role of OS in cervical ripening. Cervical ripening is a complex process being a derivative of enzymatic breakdown, inflammatory response, and endocrine regulation. There are many studies that thoroughly investigate the influence of specific factors on cervical tissue. However, it has not yet been determined whether there is one prevailing element or pathway responsible for initiating the complex of biochemical changes occurring in cervical tissue. We suggest that oxidative stress and reactive oxygen species may be one of the predominant factors in initiating and regulating a number of biochemical pathways that occur during the cervical remodeling process. Undoubtedly, pregnancy and labor are stages related to increased oxidative stress, resulting in high amounts of ROS and RNS. This is mainly due to increased metabolic requirements and oxygen consumption, which continue to increase during pregnancy. As mentioned above, oxidative stress has a multidirectional direct and indirect effect on the pregnant cervix. 

Reactive oxygen species are potent apoptosis inducers—as mentioned above, they have the ability to trigger most of the apoptotic pathways. Additionally, they actively participate in apoptosis regulation. The gestation time is specific to the human species. This may suggest that the death of the cervical cells may be initially programmed as a normal physiological event. Upon the annihilation of cervical cells, a subsequent infiltration of leukocytes occurs. In turn, inflammatory cells release inflammatory mediators and proteolytic enzymes such as inflammatory cytokines and MMPs that further degrade the cervical stroma and enhance inflammatory response. This may suggest that programmed apoptosis of cervical cells, which can be triggered by ROS, strongly influences biochemical and molecular cascades of cervical ripening.

Cervical ripening corresponds in many aspects to the inflammatory process. Inflammatory cytokines, NO, and prostaglandins are key players responsible for cervical tissue remodeling. ROS can strongly upregulate inflammatory reactions in the cervix by stimulating NF-κB and p38MAPK action. Subsequently, the inflammatory response amplifies, leading to cervical extracellular matrix remodeling. As reported previously, oxidative stress-induced p38MAPK activation has been shown to contribute to sterile inflammation occurring in cervical tissue. This suggests that besides other known ripening mediators, the p38MAPK cascade may significantly influence cervical tissue remodeling. Therefore, we believe that inhibiting the p38MAPK signaling cascade may be a potentially novel approach to preventing adverse pregnancy outcomes related to oxidative stress, such as preterm birth. However, this issue requires further extensive research. 

Nitric oxide is responsible for a variety of actions in human pregnancy. NO, not only directly stimulates MMPs action but also indirectly enhances inflammatory response via sustaining continuous NF-κB activation. Moreover, NO can be actively involved in cervical cell apoptosis. Additionally, it is involved in maintaining the proper course of pregnancy by providing uterine quiescence during pregnancy and preserving low vascular flow resistance in the umbilical vessels. Of all the factors influencing cervical ripening, nitric oxide may be the most important due to its multi-level action. As mentioned above, the local application of NO donor can significantly induce cervical ripening [91]. Additionally, it turns out that the application of NO donors is associated with a significant reduction in the percentage of uterine hyperstimulation compared to the use of prostaglandin analogs which are broadly used to labor induction nowadays [108]. This may suggest that nitric oxide donors may be used as an effective cervical ripening agent. However, the above statements require further extensive studies with a suitable NO donor which can be safely administered.

Due to the multifactorial nature of cervical changes, it can be challenging to predict the occurrence of cervical ripening from one biological marker. An emerging topic that can help with this issue is machine learning. This method, which is currently gaining publicity, is used to predict the occurrence of cervical tumors, liver tumors, and COVID-19 infection. Thanks to the development of a machine learning platform, it is possible to establish a predictive model for the occurrence of specific diseases [109,110]. We believe that the use of similar methods could be effective in predicting the occurrence of cervical ripening. Estimating the risk of the occurrence of cervical ripening might be extremely helpful in predicting possible premature birth. However, this issue requires further, extensive research that takes into account the multifactorial nature of changes in the cervix depending on the gestational age.

Considering the above, oxidative stress plays a significant role in most of the molecular and biochemical pathways in the cervix of the pregnant woman.

A complete understanding of the entire cervical ripening process seems crucial in developing new effective methods to cope with adverse pregnancy outcomes such as preterm labor.

## Data Availability

Not applicable.
